# 1-[4-(2-Fur­yl)but-3-en-2-yl­idene]-2-(2-nitro­phen­yl)hydrazine

**DOI:** 10.1107/S1600536809031638

**Published:** 2009-08-15

**Authors:** Zhi-gang Yin, Heng-yu Qian, Chun-xia Zhang, Zhi-qiang Yao

**Affiliations:** a Key Laboratory of Surface and Interface Science of Henan, School of Materials & Chemical Engineering, Zhengzhou University of Light Industry, Zhengzhou 450002, People’s Republic of China

## Abstract

In the title Schiff base compound, C_14_H_13_N_3_O_3_, the furan and benzene rings are oriented at a dihedral angle of 10.24 (13)°. Intra­molecular N—H⋯O hydrogen bonding is observed between the imino and nitro groups.

## Related literature

For applications of Schiff base compounds, see: Okabe *et al.* (1993 ▶).
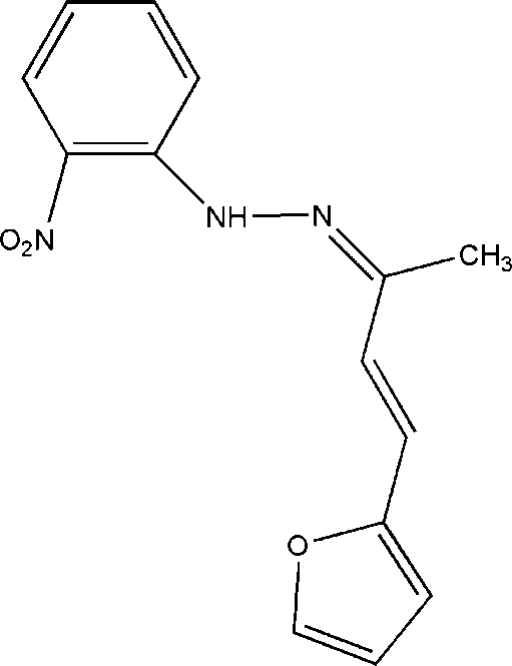


## Experimental

### 

#### Crystal data

C_14_H_13_N_3_O_3_
*M* *_r_* = 271.27
Triclinic, 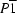

*a* = 8.2261 (2) Å
*b* = 9.0200 (2) Å
*c* = 9.1027 (2) Å
α = 89.166 (2)°
β = 77.549 (2)°
γ = 80.250 (2)°
*V* = 649.83 (3) Å^3^
*Z* = 2
Mo *K*α radiation
μ = 0.1 mm^−1^
*T* = 296 K
0.12 × 0.10 × 0.07 mm

#### Data collection

Bruker SMART CCD area-detector diffractometer
Absorption correction: none
9374 measured reflections
2687 independent reflections
1285 reflections with *I* > 2σ(*I*)
*R*_int_ = 0.039

#### Refinement

*R*[*F* ^2^ > 2σ(*F* ^2^)] = 0.050
*wR*(*F* ^2^) = 0.136
*S* = 0.90
2687 reflections
182 parameters
H-atom parameters constrained
Δρ_max_ = 0.19 e Å^−3^
Δρ_min_ = −0.17 e Å^−3^

### 

Data collection: *SMART* (Bruker, 1998 ▶); cell refinement: *SAINT* (Bruker, 1998 ▶); data reduction: *SAINT*; program(s) used to solve structure: *SHELXS97* (Sheldrick, 2008 ▶); program(s) used to refine structure: *SHELXL97* (Sheldrick, 2008 ▶); molecular graphics: *ORTEP-3 for Windows* (Farrugia, 1997 ▶); software used to prepare material for publication: *WinGX* (Farrugia, 1999 ▶).

## Supplementary Material

Crystal structure: contains datablocks global, I. DOI: 10.1107/S1600536809031638/xu2582sup1.cif

Structure factors: contains datablocks I. DOI: 10.1107/S1600536809031638/xu2582Isup2.hkl

Additional supplementary materials:  crystallographic information; 3D view; checkCIF report

## Figures and Tables

**Table 1:** Hydrogen-bond geometry (Å, °)

*D*—H⋯*A*	*D*—H	H⋯*A*	*D*⋯*A*	*D*—H⋯*A*
N2—H2*A*⋯O2	0.86	2.00	2.611 (2)	127

**Fig. 1.**
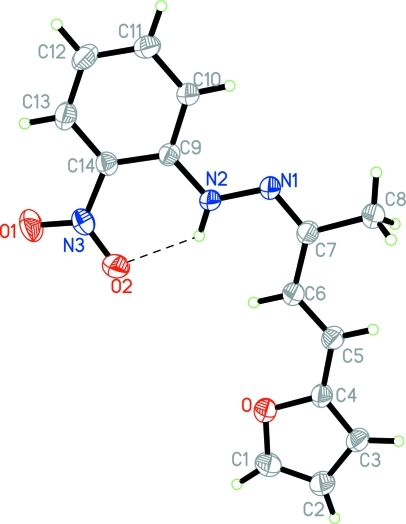
The molecular structure of the title compound. Displacement ellipsoids are drawn at the 30% probability level. Hydrogen bond is shown in dashed line.

## supplementary crystallographic information

### Comment

4-Nitrophenylhydrazine has applications in organic synthesis, and some of its derivatives have been shown to be potentially DNA-damaging and mutagenic agents (Okabe *et al.*, 1993). As part of our work, the title compound (I) is synthesized in our group, and its structure is reported here (Fig. 1).

The molecular structure is non-planar, there is a dihedral angle of 9.19 (9)° between the benzene ring and the N2/N1/C7/C6/C5 plane, while the N2/N1/C7/C6/C5 planar and the furyl ring is nearly planar, making a dihedral angle of 4.26 (11)°. The furan and benzene rings are oriented at a dihedral angle of 10.24 (13)°. Intramolecular N—H···O hydrogen bonding is present between imino and nitro groups (Table 1).

### Experimental

2-Nitrophenylhydrazine (1 mmol, 0.153 g) was dissolved in anhydrous ethanol (15 ml). The mixture was stirred for several min at 351 K, then the furylideneacetone (1 mmol, 0.136 g) in ethanol (8 ml) was added dropwise and the mixture was stirred at refluxing temperature for 2 h. The product was isolated and recrystallized from methanol, red single crystals of (I) were obtained after 3 d.

### Refinement

All H atoms were positioned geometrically and refined as riding with C—H = 0.93 (aromatic), 0.96 Å (methyl) and N—H = 0.86 Å, and refined in riding mode with *U*_iso_(H) = 1.5*U*_eq_(C) for methyl H atoms and *U*_iso_(H) = 1.2*U*_eq_(C,*N*) for the others.

### Crystal data

**Table:**

C_14_H_13_N_3_O_3_	*Z* = 2
*M**_r_* = 271.27	*F*(000) = 284
Triclinic, *P*1	*D*_x_ = 1.386 Mg m^−^^3^
Hall symbol: -P 1	Mo *K*α radiation, λ = 0.71073 Å
*a* = 8.2261 (2) Å	Cell parameters from 1117 reflections
*b* = 9.0200 (2) Å	θ = 2.3–26.5°
*c* = 9.1027 (2) Å	µ = 0.1 mm^−^^1^
α = 89.166 (2)°	*T* = 296 K
β = 77.549 (2)°	Plate, red
γ = 80.250 (2)°	0.12 × 0.10 × 0.07 mm
*V* = 649.83 (3) Å^3^	

### Data collection

**Table:**

Bruker SMART CCD area-detector diffractometer	1285 reflections with *I* > 2σ(*I*)
Radiation source: fine-focus sealed tube	*R*_int_ = 0.039
graphite	θ_max_ = 26.5°, θ_min_ = 2.3°
ω scans	*h* = −10→10
9374 measured reflections	*k* = −11→10
2687 independent reflections	*l* = −11→10

### Refinement

**Table:**

Refinement on *F*^2^	Primary atom site location: structure-invariant direct methods
Least-squares matrix: full	Secondary atom site location: difference Fourier map
*R*[*F*^2^ > 2σ(*F*^2^)] = 0.050	Hydrogen site location: inferred from neighbouring sites
*wR*(*F*^2^) = 0.136	H-atom parameters constrained
*S* = 0.90	*w* = 1/[σ^2^(*F*_o_^2^) + (0.0668*P*)^2^] where *P* = (*F*_o_^2^ + 2*F*_c_^2^)/3
2687 reflections	(Δ/σ)_max_ < 0.001
182 parameters	Δρ_max_ = 0.19 e Å^−^^3^
0 restraints	Δρ_min_ = −0.17 e Å^−^^3^

### Special details

**Table:**

Geometry. All e.s.d.'s (except the e.s.d. in the dihedral angle between two l.s. planes) are estimated using the full covariance matrix. The cell e.s.d.'s are taken into account individually in the estimation of e.s.d.'s in distances, angles and torsion angles; correlations between e.s.d.'s in cell parameters are only used when they are defined by crystal symmetry. An approximate (isotropic) treatment of cell e.s.d.'s is used for estimating e.s.d.'s involving l.s. planes.
Refinement. Refinement of *F*^2^ against ALL reflections. The weighted *R*-factor *wR* and goodness of fit *S* are based on *F*^2^, conventional *R*-factors *R* are based on *F*, with *F* set to zero for negative *F*^2^. The threshold expression of *F*^2^ > σ(*F*^2^) is used only for calculating *R*-factors(gt) *etc*. and is not relevant to the choice of reflections for refinement. *R*-factors based on *F*^2^ are statistically about twice as large as those based on *F*, and *R*- factors based on ALL data will be even larger.

### Fractional atomic coordinates and isotropic or equivalent isotropic displacement parameters (Å^2^)

**Table:**

	*x*	*y*	*z*	*U*_iso_*/*U*_eq_	
N2	0.15950 (19)	0.59694 (17)	0.52496 (19)	0.0473 (5)	
H2A	0.1972	0.5044	0.5403	0.057*	
N1	0.2372 (2)	0.66928 (18)	0.40298 (19)	0.0470 (5)	
O	0.58672 (18)	0.12781 (16)	0.35428 (17)	0.0599 (5)	
C9	0.0217 (2)	0.6737 (2)	0.6216 (2)	0.0417 (5)	
C14	−0.0559 (2)	0.6146 (2)	0.7579 (2)	0.0453 (6)	
N3	−0.0014 (2)	0.4629 (2)	0.8002 (2)	0.0556 (5)	
C7	0.3716 (2)	0.5962 (2)	0.3164 (2)	0.0461 (6)	
O1	−0.0875 (2)	0.41053 (18)	0.9101 (2)	0.0751 (5)	
C6	0.4454 (2)	0.4401 (2)	0.3302 (2)	0.0481 (6)	
H6A	0.3927	0.3866	0.4093	0.058*	
O2	0.1291 (2)	0.38750 (17)	0.72528 (19)	0.0727 (5)	
C10	−0.0499 (3)	0.8183 (2)	0.5884 (3)	0.0519 (6)	
H10A	−0.0067	0.8594	0.4969	0.062*	
C3	0.7845 (3)	0.1262 (2)	0.1462 (3)	0.0581 (7)	
H3A	0.8526	0.1572	0.0600	0.070*	
C4	0.6560 (2)	0.2129 (2)	0.2398 (2)	0.0480 (6)	
C11	−0.1819 (3)	0.9011 (2)	0.6869 (3)	0.0606 (7)	
H11A	−0.2263	0.9974	0.6615	0.073*	
C5	0.5839 (2)	0.3679 (2)	0.2372 (2)	0.0486 (6)	
H5A	0.6390	0.4249	0.1628	0.058*	
C8	0.4503 (3)	0.6843 (2)	0.1883 (3)	0.0645 (7)	
H8A	0.3815	0.7816	0.1884	0.097*	
H8B	0.4589	0.6319	0.0952	0.097*	
H8C	0.5610	0.6961	0.1990	0.097*	
C13	−0.1892 (3)	0.7005 (3)	0.8576 (3)	0.0588 (7)	
H13A	−0.2368	0.6600	0.9479	0.071*	
C12	−0.2507 (3)	0.8442 (3)	0.8237 (3)	0.0633 (7)	
H12A	−0.3376	0.9029	0.8917	0.076*	
C2	0.7968 (3)	−0.0203 (3)	0.2030 (3)	0.0648 (7)	
H2B	0.8740	−0.1046	0.1615	0.078*	
C1	0.6766 (3)	−0.0147 (3)	0.3274 (3)	0.0659 (7)	
H1B	0.6563	−0.0965	0.3878	0.079*	

### Atomic displacement parameters (Å^2^)

**Table:**

	*U*^11^	*U*^22^	*U*^33^	*U*^12^	*U*^13^	*U*^23^
N2	0.0478 (10)	0.0362 (10)	0.0488 (12)	−0.0004 (8)	0.0038 (9)	0.0049 (9)
N1	0.0481 (10)	0.0431 (10)	0.0440 (11)	−0.0059 (8)	0.0013 (9)	0.0049 (9)
O	0.0627 (10)	0.0535 (10)	0.0539 (11)	−0.0022 (8)	0.0025 (8)	0.0052 (8)
C9	0.0403 (11)	0.0419 (12)	0.0415 (14)	−0.0082 (9)	−0.0047 (10)	−0.0008 (10)
C14	0.0456 (12)	0.0465 (13)	0.0440 (14)	−0.0111 (10)	−0.0078 (11)	0.0050 (11)
N3	0.0626 (13)	0.0520 (12)	0.0534 (13)	−0.0170 (10)	−0.0103 (11)	0.0130 (10)
C7	0.0441 (12)	0.0452 (13)	0.0458 (14)	−0.0076 (10)	−0.0023 (10)	−0.0006 (11)
O1	0.0893 (12)	0.0741 (12)	0.0618 (12)	−0.0307 (10)	−0.0041 (10)	0.0247 (9)
C6	0.0484 (12)	0.0458 (13)	0.0460 (14)	−0.0057 (10)	−0.0030 (11)	0.0015 (11)
O2	0.0690 (11)	0.0546 (10)	0.0825 (13)	0.0023 (8)	−0.0015 (10)	0.0187 (9)
C10	0.0535 (13)	0.0425 (13)	0.0514 (15)	−0.0020 (10)	0.0017 (11)	0.0051 (11)
C3	0.0548 (14)	0.0449 (14)	0.0628 (17)	−0.0019 (11)	0.0080 (12)	0.0013 (12)
C4	0.0465 (13)	0.0467 (13)	0.0480 (15)	−0.0096 (10)	−0.0028 (11)	0.0033 (12)
C11	0.0580 (14)	0.0459 (14)	0.0684 (18)	0.0011 (11)	−0.0007 (13)	−0.0017 (13)
C5	0.0470 (12)	0.0457 (13)	0.0499 (15)	−0.0082 (10)	−0.0034 (11)	−0.0021 (11)
C8	0.0672 (15)	0.0504 (14)	0.0618 (17)	−0.0025 (12)	0.0106 (13)	0.0065 (13)
C13	0.0563 (14)	0.0671 (16)	0.0476 (15)	−0.0122 (12)	0.0020 (12)	0.0023 (13)
C12	0.0538 (14)	0.0635 (16)	0.0607 (17)	−0.0011 (12)	0.0072 (12)	−0.0124 (14)
C2	0.0687 (16)	0.0495 (15)	0.0655 (18)	0.0015 (12)	0.0003 (14)	−0.0011 (13)
C1	0.0757 (17)	0.0452 (14)	0.0707 (18)	−0.0023 (12)	−0.0089 (15)	0.0070 (13)

### Geometric parameters (Å, °)

**Table:**

N2—C9	1.364 (2)	C10—H10A	0.9300
N2—N1	1.372 (2)	C3—C4	1.344 (3)
N2—H2A	0.8600	C3—C2	1.407 (3)
N1—C7	1.293 (2)	C3—H3A	0.9300
O—C4	1.367 (2)	C4—C5	1.425 (3)
O—C1	1.367 (2)	C11—C12	1.382 (3)
C9—C10	1.394 (3)	C11—H11A	0.9300
C9—C14	1.409 (3)	C5—H5A	0.9300
C14—C13	1.389 (3)	C8—H8A	0.9600
C14—N3	1.440 (3)	C8—H8B	0.9600
N3—O1	1.232 (2)	C8—H8C	0.9600
N3—O2	1.238 (2)	C13—C12	1.366 (3)
C7—C6	1.451 (3)	C13—H13A	0.9300
C7—C8	1.494 (3)	C12—H12A	0.9300
C6—C5	1.337 (2)	C2—C1	1.328 (3)
C6—H6A	0.9300	C2—H2B	0.9300
C10—C11	1.366 (3)	C1—H1B	0.9300
			
C9—N2—N1	119.11 (16)	C3—C4—C5	132.1 (2)
C9—N2—H2A	120.4	O—C4—C5	118.40 (18)
N1—N2—H2A	120.4	C10—C11—C12	121.2 (2)
C7—N1—N2	118.00 (17)	C10—C11—H11A	119.4
C4—O—C1	106.09 (16)	C12—C11—H11A	119.4
N2—C9—C10	120.34 (19)	C6—C5—C4	127.0 (2)
N2—C9—C14	123.30 (18)	C6—C5—H5A	116.5
C10—C9—C14	116.36 (18)	C4—C5—H5A	116.5
C13—C14—C9	121.3 (2)	C7—C8—H8A	109.5
C13—C14—N3	117.0 (2)	C7—C8—H8B	109.5
C9—C14—N3	121.75 (18)	H8A—C8—H8B	109.5
O1—N3—O2	121.45 (19)	C7—C8—H8C	109.5
O1—N3—C14	118.91 (19)	H8A—C8—H8C	109.5
O2—N3—C14	119.64 (18)	H8B—C8—H8C	109.5
N1—C7—C6	126.3 (2)	C12—C13—C14	120.4 (2)
N1—C7—C8	114.50 (19)	C12—C13—H13A	119.8
C6—C7—C8	119.16 (18)	C14—C13—H13A	119.8
C5—C6—C7	124.5 (2)	C13—C12—C11	119.0 (2)
C5—C6—H6A	117.7	C13—C12—H12A	120.5
C7—C6—H6A	117.7	C11—C12—H12A	120.5
C11—C10—C9	121.6 (2)	C1—C2—C3	106.61 (19)
C11—C10—H10A	119.2	C1—C2—H2B	126.7
C9—C10—H10A	119.2	C3—C2—H2B	126.7
C4—C3—C2	107.2 (2)	C2—C1—O	110.6 (2)
C4—C3—H3A	126.4	C2—C1—H1B	124.7
C2—C3—H3A	126.4	O—C1—H1B	124.7
C3—C4—O	109.46 (18)		
			
C9—N2—N1—C7	177.25 (18)	C14—C9—C10—C11	3.7 (3)
N1—N2—C9—C10	6.7 (3)	C2—C3—C4—O	0.4 (3)
N1—N2—C9—C14	−173.15 (18)	C2—C3—C4—C5	−178.4 (2)
N2—C9—C14—C13	175.70 (19)	C1—O—C4—C3	−0.3 (3)
C10—C9—C14—C13	−4.2 (3)	C1—O—C4—C5	178.63 (19)
N2—C9—C14—N3	−4.5 (3)	C9—C10—C11—C12	−0.4 (4)
C10—C9—C14—N3	175.66 (18)	C7—C6—C5—C4	−175.9 (2)
C13—C14—N3—O1	9.4 (3)	C3—C4—C5—C6	173.7 (2)
C9—C14—N3—O1	−170.41 (19)	O—C4—C5—C6	−5.0 (3)
C13—C14—N3—O2	−171.21 (19)	C9—C14—C13—C12	1.5 (3)
C9—C14—N3—O2	8.9 (3)	N3—C14—C13—C12	−178.4 (2)
N2—N1—C7—C6	3.5 (3)	C14—C13—C12—C11	2.0 (4)
N2—N1—C7—C8	−178.71 (18)	C10—C11—C12—C13	−2.5 (4)
N1—C7—C6—C5	179.3 (2)	C4—C3—C2—C1	−0.2 (3)
C8—C7—C6—C5	1.6 (3)	C3—C2—C1—O	0.0 (3)
N2—C9—C10—C11	−176.2 (2)	C4—O—C1—C2	0.2 (3)

### Hydrogen-bond geometry (Å, °)

**Table:**

*D*—H···*A*	*D*—H	H···*A*	*D*···*A*	*D*—H···*A*
N2—H2A···O2	0.86	2.00	2.611 (2)	127
